# Supplementary figures and images for: Use of Epivolve phage display to generate a monoclonal antibody with opsonic activity directed against a subdominant epitope on extracellular loop 4 of Treponema pallidum BamA (TP0326)
Source: Front Immunol. 2023 Aug 22;14:1222267. doi: 10.3389/fimmu.2023.1222267 (PMC10478084; doi:10.3389/fimmu.2023.1222267)

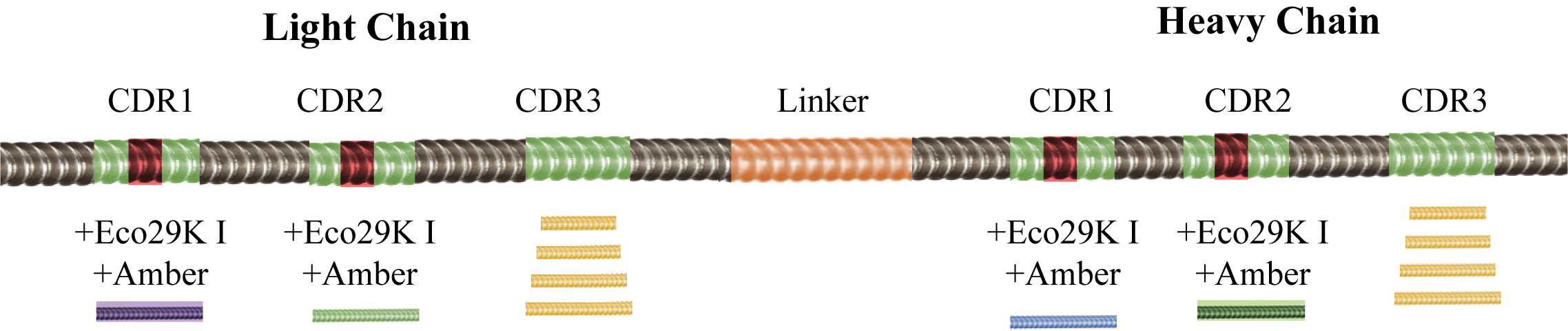

Supplement: Supplementary Figure 1 — Library vector template. The parental vector was modified to contain four Eco29kI restriction endonuclease sites and amber (5’-TAG-3’) stop codons within the CDRs targeted for mutagenesis. For library generation, amino acid stretches varying from six to 22 residues were incorporated into LC and HC CDR3s. Colored bars represent designate primer binding sites. [file Image_1.tif]

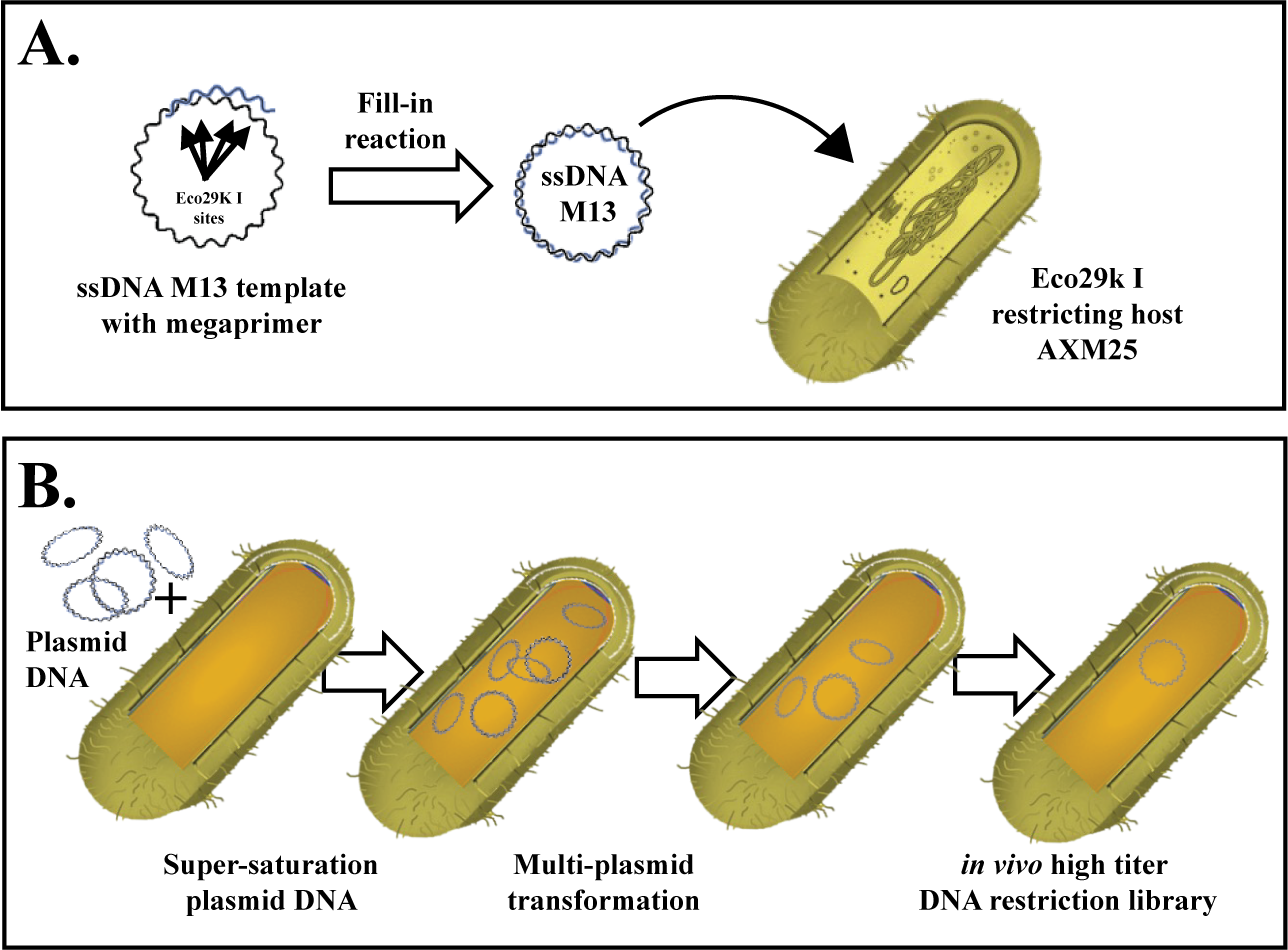

Supplement: Supplementary Figure 2 — Production of high-titer, fully recombinant Ab libraries. (A) In vivo restriction using E. coli AXE688 [TG1 (eco29KI.RM)] to produce recombinant libraries (21, 23). The AXM mutagenesis method (21, 23) involves the use of PCR under specific conditions to create a mutated DNA fragment by promoting nucleotide misincorporation during DNA synthesis. In this PCR reaction, one of the primers contains at least three phosphorothioate linkages at its 5′ end. After PCR, the resulting product is treated with a 5′ to 3′ exonuclease, which selectively removes the strand synthesized with the non-modified primer, leaving behind a single-stranded DNA fragment. This single-stranded DNA fragment acts as a megaprimer in a Kunkel-like mutagenesis reaction, where it primes DNA synthesis on a circular, single-stranded template that has been uracilated. This reaction biases nucleotide base-changes between the megaprimer and the uracilated DNA sequence, favoring the in vitro synthesized megaprimer. Parental plasmids carrying Eco29kI sites within the complementarity determining regions (CDRs) of the scFv are cleaved by Eco29kI expressed in the AXE688 cells. (B) In vivo selection using saturating DNA. Super-saturating concentrations of plasmid DNA were used generate large recombinant libraries. Competent cells take up multiple plasmids under DNA saturating conditions using AXE688, thereby resulting in transformed cells with a higher proportion of totally recombinant clones. [file Image_2.tif]

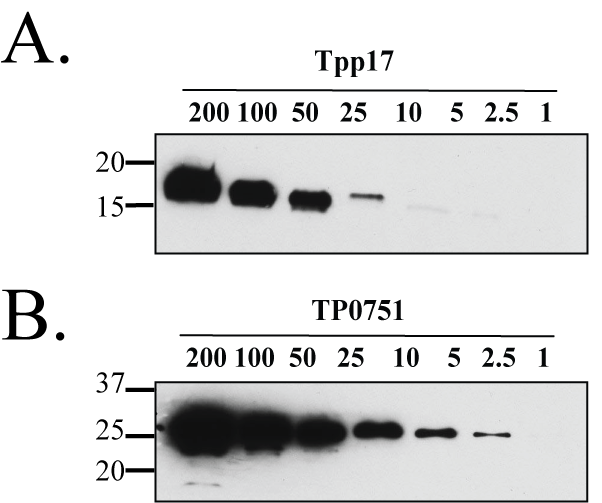

Supplement: Supplementary Figure 3 — Reactivity of mouse Tpp17 and TP0751 antisera. Reactivity of sera (diluted 1:1,000) from mice hyperimmunized with Tpp17 or TP0751 by immunoblot analysis against graded nanogram amounts of (A) Tpp17 or (B) TP0751. [file Image_3.tif]
